# Bidirectional Clinical Interactions among Exacerbations and Comorbidities in COPD: A Narrative Review

**DOI:** 10.1055/a-2835-0340

**Published:** 2026-04-10

**Authors:** Daphne E. M. Peerlings, Sarah Houben-Wilke, Sami O. Simons, Anne E. Ioannides, Jennifer K. Quint, Frits M. E. Franssen

**Affiliations:** 1 Department of Research and Development, Ciro, Horn, the Netherlands; 2 Department of Respiratory Medicine, NUTRIM Research Institute of Nutrition and Translational Research in Metabolism, Faculty of Health Medicine and Life Sciences, Maastricht University, Maastricht, the Netherlands; 3 Department of Respiratory Medicine, Maastricht University Medical Centre, Maastricht, the Netherlands; 4 Department of Primary Care and Public Health, Imperial College London, London, United Kingdom

**Keywords:** exacerbations, COPD, comorbidity, interorgan crosstalk, multimorbidity, differential diagnosis

## Abstract

Acute deteriorations of respiratory symptoms in people with chronic obstructive pulmonary disease (COPD), known as exacerbations, worsen COPD severity (e.g., speed up lung function decline), and increase hospital admissions, healthcare costs, and mortality risk. The prevention, diagnosis, and treatment of exacerbations remain challenging due to the heterogeneous nature of these events. This complexity is further compounded by the high prevalence of multiple comorbidities and incompletely understood underlying mechanisms. Exacerbations of COPD and comorbidities are linked through bidirectional relationships, characterized by mutual adverse impacts, overlapping clinical manifestations, and increased susceptibility to the other condition. The identification and management of comorbidities are pivotal for effective disease management. Although current clinical frameworks, that is, models that integrate clinical features and biomarker-based identification of exacerbations to guide risk stratification and management, represent promising approaches to improve patient outcomes, multimorbidity is insufficiently incorporated. This narrative review provides an overview of the complex clinical associations of comorbidities in COPD, with a particular focus on exacerbations. It highlights differences in comorbidity prevalence among exacerbators, explores clinical interrelationships, and underscores the importance of multimorbidity-oriented management.

## Introduction

Chronic obstructive pulmonary disease (COPD) is a heterogeneous and frequently multimorbid disease, affecting approximately 480 million individuals worldwide (10.6%) in 2020 and estimated to reach 592 million individuals (9.5%) by 2050, a 23.3% increase. 1 Since 2023, COPD has been the third leading cause of death worldwide, 2 3 underlying the substantial global burden of the disease. 4 5 6 7 Up to 97.7% of patients with COPD exhibit at least one comorbidity, defined as the co-occurrence of another chronic condition, and more than half (52–54%) exhibit four or more comorbidities. 8 9 10

In literature, comorbidities in COPD are commonly clustered into cardiovascular disease (CVD: e.g., hypertension, coronary artery disease, ischemic heart disease and heart failure), metabolic (e.g., dyslipidemia, metabolic syndrome, diabetes mellitus, and obesity), psychological (e.g., anxiety and depression), cachectic (e.g., underweight, low muscle mass, sarcopenia, and osteoporosis), and other conditions (e.g., malignancy, osteoarthritis, sleep apnea, and gastroesophageal reflux). 8 11 12 13 COPD and its comorbidities share several risk factors, including smoking, environmental pollution, genetic predisposition, abnormal lung development, and infections. 3 4 8 Additionally, common pathogenic pathways have been identified, such as chronic (systemic) inflammation, hypoxia, and oxidative stress. 10 11 14 These shared mechanisms may give rise to bidirectional interactions between different organs (interorgan crosstalk), thereby reinforcing the understanding of COPD as a systemic disease. 3 11

Such bidirectional interactions are especially challenging in the context of exacerbations in COPD, during which comorbidities, and vice versa, may mimic, aggravate, precipitate, or increase the risk of one another. 3 15 Approximately half of all individuals with COPD experience at least one exacerbation annually, with 12.6 to 15.2% experiencing a severe event. 16 17 Exacerbations contribute to disease progression by accelerating lung function decline, reducing quality of life, worsening comorbidity risk and associated health outcomes, and increasing hospital (re-)admissions, mortality rates, and health care costs. 3 15 18 19 20 21 22 23 24 Consequently, prevention and timely treatment of both exacerbations and comorbidities are central components of COPD management.

Interactions among comorbidities also occur independently of COPD. For instance, a meta-analysis of 21 studies showed that individuals with metabolic syndrome have an increased relative CVD risk and CVD-related mortality. 25 In addition, given that (1) both COPD itself 26 and exacerbations thereof 27 are associated with CVD, and (2) that there are interactions occurring between non-COPD comorbidities themselves and CVD, effective management of complex clinical cases becomes imperative, particularly when COPD patients exacerbate. 27

The focus has shifted from considering individual comorbidities to a multimorbidity or syndemic perspective, which acknowledges the clustering and interaction of coexisting conditions. Accordingly, this narrative review summarizes current knowledge on the clinical associations of comorbidities and exacerbations of COPD. It examines differences in comorbidity prevalence between high-risk exacerbators and patients without stratification by exacerbation risk, explores clinical associations both between exacerbations and comorbidities and among comorbidities themselves, and discusses unmet needs in effective disease management.

## Prevalences of Comorbidities in Patients with Exacerbations of COPD

**Fig. 1:**
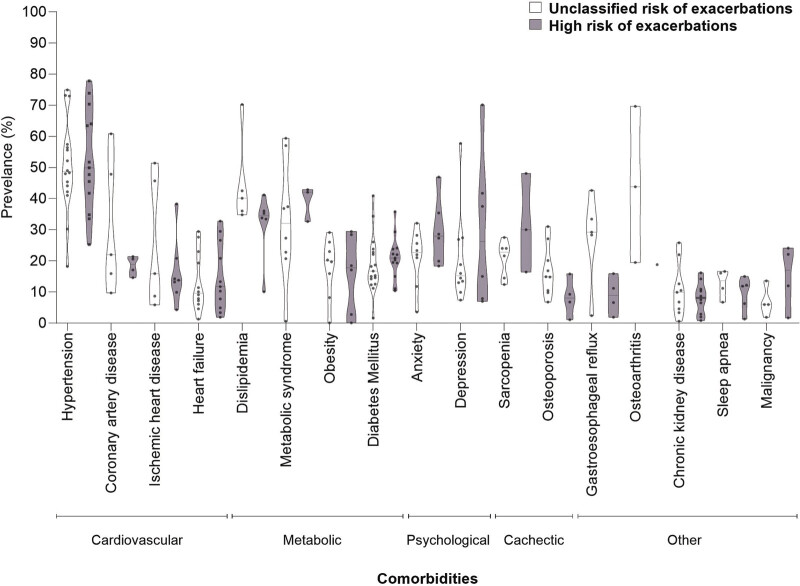
Prevalence rankings (median values with minimum–maximum %) of comorbidities in study cohorts, including patients with COPD and unclassified risk of exacerbations (white) and high risk of exacerbations (colored). 8 9 27 29 30 31 32 33 34 35 36 37 38 39 40 41 42 43 44 45 46 47 48 49 50 51 52 53 54 55 56 57 58 59 60 61 62 63 64 65 66 67 68 69 70 71 72 73 74 75 76 77 78 79

Comorbidity prevalence in COPD is often reported for the overall population without distinguishing patients by exacerbation risk (i.e., unclassified risk of exacerbations), despite the well-recognized heterogeneity. However, growing evidence indicates that certain comorbidities are either more or less prevalent among patients at high risk of exacerbations (i.e., ≥1–2 events per year and/or severe exacerbations, depending on the GOLD reports from 2011 to 2025 or the 2026 update 3 28 ). An overview of these comorbidity prevalence patterns is illustrated in Fig. 1 and Supplemental Table S1 (available in the online version only). 8 9 27 29 30 31 32 33 34 35 36 37 38 39 40 41 42 43 44 45 46 47 48 49 50 51 52 53 54 55 56 57 58 59 60 61 62 63 64 65 66 67 68 69 70 71 72 73 74 75 76 77 78 79

Although CVD and metabolic comorbidities are predominant in both groups, conditions such as anxiety, depression, sarcopenia, heart failure, and malignancy appear to be more prevalent amongst COPD patients at high risk of exacerbations. Dyslipidemia, osteoarthritis, coronary artery disease, metabolic syndrome, gastroesophageal reflux, osteoporosis, and chronic kidney disease, however, appear to be more prevalent across the COPD population in general. 8 9 27 29 30 31 32 33 34 35 36 37 38 39 40 41 42 43 44 45 46 47 48 49 50 51 52 53 54 55 56 57 58 59 60 61 62 63 64 65 66 67 68 69 70 71 72 73 74 75 76 77 78 79

The marked variation in comorbidity prevalence across studies may be explained by heterogeneity in study populations, inclusion criteria, study periods, assessment methods, diagnostic definitions, and geographic differences across Asia, Europe, and America. 80 Alternatively, these differences may reflect underlying patient vulnerability or distinct disease mechanisms. Furthermore, overlapping disease profiles further complicate accurate diagnosis. For example, increased cough and shortness of breath due to congestive heart failure overlap with the symptoms and definition of exacerbations in COPD. 3 15 In addition, it is estimated that 15 to 19% of all exacerbations are caused by pulmonary embolism, pneumonia, or heart failure. 81 82 Comorbidities or exacerbations of COPD may therefore act as mutual determinants, representing a multimorbidity network, a concept that is further explored in the following paragraphs.

## Comorbidities as Modifiers of Exacerbation Burden: Toward a Multimorbidity Network During Exacerbations of COPD

Comorbidities are increasingly recognized as important modifiers of exacerbation risk and clinical outcomes. Several studies have reported significant associations between specific comorbid conditions and the frequency, severity, and prognosis of exacerbations; a detailed overview of these associations is provided in Table 1 . 9 41 56 57 60 70 83 84 85 86 87 88 89 90

**Table 1:** Associations between comorbidities and clinical outcomes in exacerbations of COPD

Comorbidities	Measure of clinical burden
Cardiovascular	1.2 (1.0–1.4); odds ratio (95% CI); ≥2 vs. < 2 ECOPD/year 9 1.2 (1.1–1.2); hazard ratio (95% CI); development of first ECOPD 9
• Hypertension	3.1 (1.6–6.2); odds ratio (95% CI); independent risk factors for predicting ECOPD 85
• Ischemic heart disease	ECOPD symptom recovery takes 5 d longer in patients with COPD and ischemic heart disease compared to those without ischemic heart disease 83
• Heart failure	1.7 (1.4–2.1); odds ratio (95% CI); ≥2 vs. < 2 ECOPD/year 9 1.4 (1.3–1.6); hazard ratio (95% CI); development of first ECOPD 9
• Coronary artery disease	1.1 (1.0–1.2); hazard ratio (95% CI); development of first ECOPD 9
Metabolic	
• Dyslipidemia	0.8 (0.7–1.0) nonstatistically ( *p* = 0.07) odds ratio (95% CI); ≥2 vs. < 2 ECOPD/year 9 0.9 (0.9–1.0); nonstatistically ( *p* = 0.07) hazard ratio (95% CI); development of first ECOPD 9
• Metabolic syndrome	2.0 (1.4–2.7); relative risk (95% CI); ECOPD risk 41
• Diabetes mellitus	0.8 (0.7–1.0); odds ratio (95% CI); ≥2 vs. < 2 ECOPD/year 9 Each 1 mmol/L increase in blood glucose was associated with a 15% (95% CI: 4–27) to 31% (95% CI: 11–55) higher relative risk of death or prolonged hospital stay 89
• Obesity	Hospitalized ECOPD with obesity (≥30) had 34–40% decreased ECOPD frequency within 5 years of follow-up, compared to a normal weight (BMI 18.5–24.9) 56 1.4 (1.1–1.8) and 1.6 (1.1–2.3); odds ratio (95% CI); moderate and severe ECOPD (respectively) 86 1.3 (1.3–1.4); odds ratio adjusted (95% CI); 4 days longer hospital stay in patients with COPD and obesity, during ECOPD, compared to nonobese 57
Psychological • Anxiety	5.9 (2.2–16.3); odds ratio (95% CI); independent risk factors for predicting exacerbations 85 1.8 (1.2–2.7); odds ratio (95% CI); influence on exacerbations 84
• Depression	1.5 (1.1–1.9); odds ratio (95% CI); ≥2 vs. < 2 exacerbations/year 9 1.3 (1.2–1.5); hazard ratio (95% CI); development of the first exacerbation 9 1.6 (1.1–2.4); odds ratio (95% CI); influence on exacerbations 84 Higher median (IQR) depression scores in frequent exacerbators (17.0 [7.0–25.0]) vs. infrequent exacerbators (12.0 [6.0–18.0]) 90 Depression scores (median [IQR]) increased from baseline (12.5 [5.0–19.0]) to exacerbation (19.5 [12.0–28.0]), without any association with exacerbation severity, duration, symptoms, or timing of next exacerbation 90
Cachectic	
• Osteoporosis	1.4 (1.1–1.8); odds ratio (95% CI); ≥2 vs. < 2 exacerbations/year 9 1.1 (1.0–1.3); hazard ratio (95% CI); development of the first exacerbation 9
• –Sarcopenia	2.4 (1.0–5.7); hazard ratio (95% CI); exacerbation risk 88
Other	
• –Malignancy	1.9 (1.3–2.7); odds ratio (95% CI); ≥2 vs. < 2 exacerbations/year 9 1.2 (1.0–1.5); hazard ratio (95% CI); first exacerbation development 9
• Chronic kidney disease	eGFR is related to exacerbation risk; every unit increase in eGFR, the exacerbation risk decreases by 0.002 units 87
• Sleep apnea	1.2 (1.2–1.2); odd ratio (95% CI); prolonged hospital stay of 4 days in patients with COPD and sleep apnea, during exacerbations, compared to no sleep apnea 70
• Osteoarthritis	0.9 (0.8–0.9); adjusted hazard ratio (95% CI); risk for hospital admission 60
• Gastro-oesophageal reflux	1.3 (1.0–1.5); odds ratio (95% CI); ≥2 vs. < 2 exacerbations/year 9 1.1 (1.0–1.2); hazard ratio (95% CI); development of the first exacerbation 9

Abbreviations: BMI, body mass index; CI, confidence interval; COPD, chronic obstructive pulmonary disease; eGFR, estimated glomerular filtration rate; IQR, interquartile range.

### Risk Assessment

Specifically, anxiety is associated with a 1.8 to 5.9 fold increased risk of exacerbations, hypertension with a 3.1 fold independent risk, and heart failure, coronary artery disease, metabolic syndrome, obesity, depression, malignancy, sleep apnea, and gastro-oesophageal reflux with a 1.1 to 2.4 fold risk. 9 41 56 57 70 84 85 86 In contrast, chronic kidney disease and osteoarthritis seem to be slightly negatively associated with exacerbation risk. 9 60 87

### Clinical Outcomes

Patients experiencing a severe exacerbation of COPD in the presence of ischemic heart disease had a 5-day longer symptom duration compared with those without this CVD comorbidity, 83 while obesity and sleep apnea were each associated with a 4-day longer hospital stay. 57 70 Interestingly, hospitalized patients with obesity may also experience a decrease in exacerbation frequency over a 5-year follow-up period. Depression scores were higher in frequent exacerbators compared with infrequent exacerbators and increased from baseline to exacerbations. However, these changes were not associated with exacerbation severity, duration, symptom burden, or time to the next exacerbation. 90 The effect of diabetes mellitus on exacerbations remains inconclusive. While some studies report no effect, others have shown that each 1 mmol/L increase in blood glucose is associated with a 15% (95% CI: 4–27) to 31% (95% CI: 11–55) higher relative risk of death or prolonged hospital stay, after adjustment for age, sex, and prior diagnosis of diabetes mellitus. 89

When interpreted within the previously described comorbidity clusters, a pattern emerges. Anxiety appears to be a strong independent predictor of exacerbations in COPD, whereas cardiovascular and some metabolic conditions (except dyslipidemia and diabetes) are mainly associated with increased exacerbation risk and prolonged recovery. Of note, patients who are anxious about exacerbations might perceive anxiety-driven respiratory sensations as exacerbation-related symptoms; however, whether this contributes to an inflation of the observed association remains to be determined.

## Exacerbations of COPD as Modifiers of Multimorbidity Burden

**Fig. 2:**
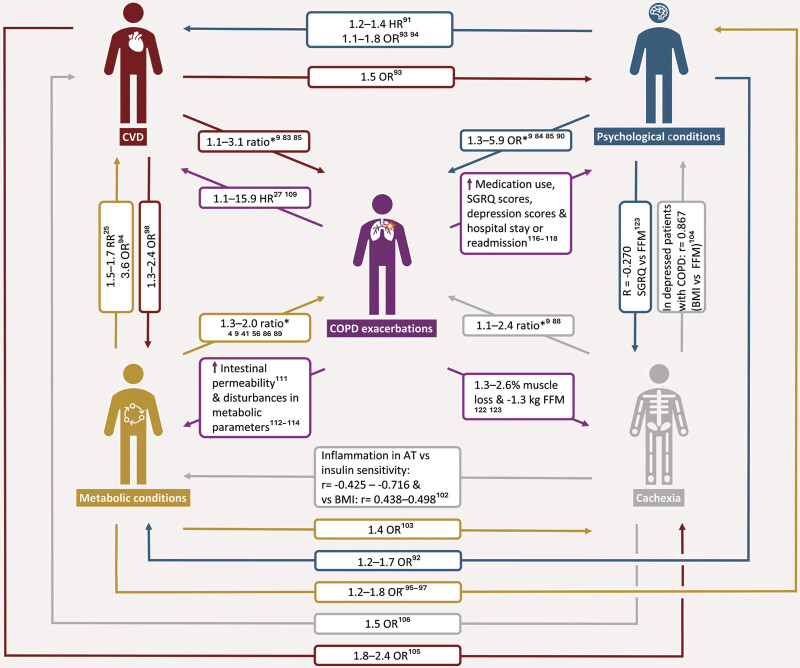
Bidirectional relationships between exacerbations of COPD and its comorbidity clusters. *See Table 1 for more details. AT, adipose tissue; BMI, body mass index; CVD, cardiovascular disease; COPD, chronic obstructive pulmonary disease; FFM, fat free mass; HR, hazard ratios; OR, odds ratios; *r* , correlation coefficient; SGRQ, St. George's Respiratory Questionnaire.

A bidirectional relationship is present between exacerbations of COPD and comorbidity clusters, with each acting as a potential modifier of the other. This relationship is further complicated by interactions among comorbidities themselves, which can affect disease presentation, complicate differential diagnosis, and amplify multimorbidity effects. A simplified network illustrating these interactions is presented in Fig. 2 , suggesting the presence of interorgan crosstalk. 25 91 92 93 94 95 96 97 98 99 100 101 102 103 104 105 106 107 108

### Exacerbations of COPD as a Modifier of Cardiovascular Disease

In a UK-specific COPD cohort, Graul et al reported that the adjusted hazard ratios (HR) for CVD were 1.8, 1.7, and 3.2 following any, moderate, and severe exacerbations, respectively, during 6 years of study follow-up, compared with no exacerbations. 27 A pronounced peak in CVD risk, 3.2 HR, was observed within the first 2 weeks after any exacerbations. From 2 to 4 weeks onward and persisting up to 1 to 6 years, CVD risk remained elevated (HR range: 1.6–2.3). Severity-specific estimates showed that CVD risk increased by 1.4 HR after moderate exacerbations and after severe exacerbations, 14.5 HR, (the highest peak for severe exacerbations) within the first 2 weeks. Thereafter, CVD risk remained elevated over time (from 2 weeks up to 6 years) after both moderate (HR range: 1.5–1.9; highest peak at 2–4 weeks) and severe (HR range: 2.4–4.5) exacerbations.

In a Canadian COPD-specific cohort, Hawkins et al similarly reported increased adjusted HR risks (HR range: 1.08–15.86) of a first severe CVD event (e.g., acute coronary syndrome, heart failure, arrhythmias) following exacerbations of any severity across multiple time points (from the first week to more than 1 year). 109 Notably, like the Graul et al study, the risk also differed across time intervals.

The differences in magnitude of reported CVD risk may be explained by methodological factors: Hawkins et al used the period prior to the first ECOPD, whereas Graul et al used those without any exacerbations. 27 109 Both studies were included in a recent meta-analysis, which also incorporated studies conducted in the Netherlands, Germany, Italy, Spain, the United States, and Japan under the exacerbations of COPD and their outcomes on the CVD umbrella. 110 Across all studies, CVD risk was consistently elevated following exacerbations, with the first weeks after an event identified as the period of highest risk, irrespective of country of origin.

### Exacerbations of COPD as a Modifier of Metabolic Conditions

Multiple studies showed that exacerbations of COPD can also influence parameters related to metabolic conditions. 89 111 112 113 During severe exacerbations of COPD, patients showed lower dietary intake (5,640 ± 2,671 kJ day ^−1^ ) than their usual intake (7,863 ± 2,005 kJ day ^−1^ ) during symptom aggravation, prior to admission, and during the first 3 days of hospitalization. No changes were observed in water compartments, fat-free mass (FFM), or body weight. Three months after admission, dietary intake had returned to usual levels, accompanied by a gain in body weight. 114 Other studies suggest that these temporary disturbances in energy metabolism are associated with increased leptin concentrations and heightened systemic inflammatory responses during exacerbations of COPD. 112 Though the effect of diabetes mellitus on exacerbations is inclusive, deteriorations in glucose metabolism are associated with worse outcomes; higher glycosylated hemoglobin (HbA1c) levels and random blood sugar levels were positively associated with the duration of hospital stay. HbA1c levels also correlated positively with the total leukocyte count, cholesterol, and body mass index (BMI), while BMI and cholesterol levels were also positively associated. In addition, a higher HbA1c level was associated with lower oxygenation, both measured by peripheral oxygen saturation and arterial partial oxygen. 113

Increased intestinal permeability is seen in patients with severe exacerbations of COPD, and has been reported as one of the mechanisms behind the metabolic derangements during exacerbations of COPD. Increased permeability facilitates bacterial translocation and systemic inflammation, impairs nutritional digestion and absorption, and thereby indirectly affects metabolic regulation. 111 115 However, as these observations are cross-sectional within the exacerbation period in COPD, it remains difficult to establish whether exacerbations themselves mediate these metabolic changes or whether preexisting metabolic dysregulation contributes to exacerbation severity.

### Exacerbations of COPD as a Modifier of Psychological Conditions

Exacerbations impose a substantial psychological burden on patients with COPD. 116 117 118 119 120 Those readmitted due to exacerbations have higher depression scores (5.0 ± 3.4 vs. 3.7 ± 3.1 points) and greater use of home medications (5.9 ± 2.2 vs. 4.5 ± 2.7), compared to patients without readmissions. This psychological and clinical impact may result in poorer health-related quality of life, as measured by the St. George's Respiratory Questionnaire (SGRQ), with higher scores indicating worse outcomes. Specifically, readmitted patients show worse scores across three SGRQ subscales: impact (social and psychological effects of disease; 46.7 ± 22.0 vs. 35.9 ± 20.6), activity (breathlessness-related limitation; 69.7 ± 19.0 vs. 59.0 ± 22.4), and symptoms (frequency and severity of respiratory complaints; 56.3 ± 19.5 vs. 45.9 ± 24.3). 116 Similar results were reported by Gudmundsson et al. 117 Higher SGRQ scores have also been observed in patients with exacerbations of COPD compared with stable COPD (60.0 ± 18.0 vs. 51.0 ± 20.0), with better processing speed index performances (cognitive function) associated with lower SGRQ scores ( *r* = −0.42) and shorter hospital stays ( *r* = −0.42) in severe exacerbators of COPD. 118 Furthermore, severe exacerbations of COPD also increased readmission risk (HR = 1.13), with readmission risk related to the interaction between SGRQ scores and the total anxiety and depression (HADS) scores ( *r* = 0.38). 117 This raises the hypothesis that the negative impact of exacerbations in COPD on health status is at least partly mediated by psychological burden.

Overall, it remains unproven whether exacerbations of COPD causally contribute to psychological conditions, despite the observed elevated burden. COPD in general has been associated with an increased risk of depression (RR: 1.7 [95% CI: 1.5–2.0]), whereas a consistent association with anxiety has not been demonstrated. 121

### Exacerbations of COPD as a Modifier of Cachexia

In the context of the cachexia comorbidity cluster, existing studies have mainly examined the relationship between exacerbations of COPD and FFM depletion or muscle loss. For instance, the exacerbation rate over time (between 3 and 5 years) was associated with skeletal muscle mass loss of 1.3 to 2.1%. 122 Moreover, patients with frequent exacerbations of COPD (annually > 1) experienced a greater loss of FFM (−1.3 kg) compared with infrequent exacerbations of COPD. 123

## Multimorbidity in the Differential Diagnoses of Exacerbations of COPD

Since comorbidities or other respiratory conditions mimic or worsen exacerbations of COPD, or vice versa, 124 differentiating between these diseases causing respiratory symptoms can be a challenge.

Current classification of exacerbation severity include at least three of the following: dyspnoea (visual analogue scale, VAS score ≥ 5), changes in sputum color or production, respiratory rate (≥24 breaths per minute), heart rate (≥95 beats per minute), hypoxaemia (arterial oxygen saturation, ≤91% and/or a change of ≥ 4%), serum C-reactive protein (CRP; ≥10 mg/L), or the presence of hypercapnia and acidosis (arterial blood gas: PaCO _2_ ≥ 46 mmHg and pH ≤ 7.34). 3 15 However, classifying severity can be complicated since these variables can also be related to concurrent heart failure, ischemic heart disease, arrhythmia, pulmonary embolism, pneumonia, bronchiectasis, asthma, interstitial lung disease (ILD), anxiety, depression, pleural effusion, lung cancer, and anaemia. 3 15 41 125 126 127 128

To aid differential diagnosis, a chest X-ray can exclude pneumonia, heart failure, pleural effusion, lung cancer, bronchiectasis, and ILD as alternative diagnoses. Computed tomography pulmonary angiogram and D-dimer concentrations can be used to exclude pulmonary embolism, and an electrocardiogram, serum troponin, and natriuretic peptides can exclude cardiac ischemia, heart failure, myocardial infarction, or arrhythmia. 3 125 129 130 131 Several respiratory disease-specific questionnaires, including the HADS, Chronic Airways Assessment Test (formerly known as the COPD Assessment Test, CAT), and SGRQ, have been developed to identify symptoms of anxiety and depression in patients with respiratory disease. 3

In clinical practice, some of these tools are not always available due to time or costs, resulting in under- or misdiagnoses. A previous study mentioned that emergency physicians missed the first diagnoses of heart failure in 62.2% of patients with a history of asthma or COPD. 129 Other studies showed that 40.1 to 49.0% of exacerbations in COPD are unreported to physicians or that 50% of physicians are not treating symptom-defined ECOPD. 82 132 133 Such under- or misdiagnoses and under- or mistreatment can lead to prolonged disease resolution, a lower quality of life, inappropriate treatments, the need for multiple or other medications, pharmacological cross-effects, antibiotic resistance, and increased healthcare costs. 3 6 34 81 125 127 134 Furthermore, multimorbidity requires specific and prompt disease management since they influence each other ( Fig. 2 ). 15

## Unmet Needs for the Management of Multimorbidity in Exacerbations of COPD: Future Guidance

**Fig. 3:**
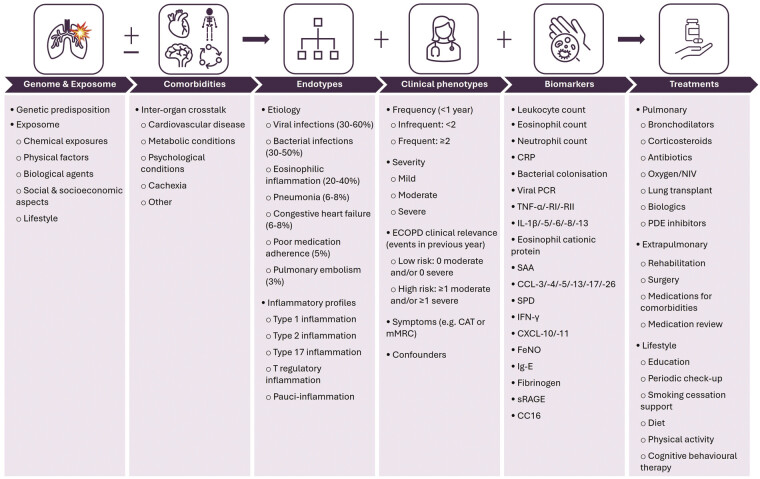
A clinical framework for the current management of exacerbations in COPD. CAT, chronic obstructive pulmonary disease assessment test; CC, club cell secretory protein; CCL, C-C motif chemokine ligand; CRP, C-reactive protein; CXCL, chemokine (C-X-C motif) ligand; FeNO, fractional exhaled nitric oxide; IFN, interferon; Ig-E, immunoglobulin-E; IL, interleukin; mMRC, modified Medical Research Council dyspnea scale; PCR, polymerase chain reaction; R, receptor; SAA, serum amyloid-A; SPD, surfactant protein D; sRAGE, soluble receptor for advanced glucation end products; TNF, tumor necrosis factor.

To optimize disease management and capture multimorbidity, further research is warranted to improve the differential diagnosis of multimorbidity and exacerbations of COPD. Key unmet needs include unravelling disease pathophysiology, enabling earlier and more accurate diagnostic tools, and developing effective therapeutic strategies. 3 4 81 135 Progress in these areas is hampered by heterogeneity and the presence of multiple comorbidities, which reflect a multilevel network of diverse biological factors and interorgan crosstalk. Framework-based modeling is the most commonly used approach 3 13 15 136 137 138 139 ; however, current strategies lack sufficient detail regarding multimorbidity-oriented management. This is illustrated in Fig. 3 , where current management incorporates multiple aspects, including the genome (genetic background), exposome (environmental exposures), endotypes (e.g., etiological and inflammatory profiles), and clinical phenotypes. Ultimately, endotypes and clinical phenotypes guide subgroup-specific treatment through the identification of relevant biomarkers.

In terms of etiology, the estimated causes of exacerbations in COPD reflect overlapping comorbidity-related factors, including 30 to 60% viral infections, 30 to 50% bacterial infections, 20 to 40% eosinophilic inflammation, 6 to 8% pneumonia, 6 to 8% congestive heart failure, 5% poor medication adherence, and 3 to 6% pulmonary embolism. 81 82 140 141 Suggested underlying inflammatory profiles have been subdivided into type 1 (neutrophil-mediated and viral-based), type 2 (eosinophil-mediated), type 17 (IL-17 driven and neutrophil attraction), regulatory T-cell-mediated (anti-inflammatory response), and pauci-inflammatory profiles. It is estimated that pauci-inflammatory profiles constitute approximately 20% of all exacerbations. 142 143 144 Nevertheless, these inflammatory responses may overlap within patients with exacerbations of COPD and among comorbidities. 145

Although numerous (inflammatory) markers have been proposed as potential biomarkers, clinically validated and specific biomarkers are generally lacking, particularly in the context of multimorbidity in exacerbations of COPD. This complicates early diagnosis, differentiation from other conditions, and guiding treatment strategies. 82 146 147 For example, biomarkers such as blood eosinophil count, immunoglobulin E (Ig-E), CRP, fibrinogen, fractional exhaled nitric oxide (FeNO), procalcitonin, interleukin (IL)-6, IL-8, soluble receptor for advanced glycation end products (sRAGE), club cell secretory protein (CC16), and surfactant protein D (SPD) have been associated with FEV1 decline, exacerbations of COPD, emphysema on imaging, hospitalization, mortality, and/or prediction of treatment response. 145 However, these biomarkers have not proven to be clinically useful for treatment decisions.

The integration of genome–exposome–driven endotypes and clinical features with biomarker profiles facilitates personalized and disease-specific treatment strategies, also called the treatable traits approach. Pulmonary, extrapulmonary, and lifestyle traits have been proposed as key subdivisions of treatable traits in COPD, although many of these traits also manifest in the context of multimorbidity. Importantly, multiple traits may co-exist within a single patient and evolve over time. Although these classifications remain insufficiently validated and lack integration with multimorbidity concepts, they provide a basis for further refinement and demonstrate potential applicability in the context of exacerbations in COPD. 3 136 148

## Conclusion

The complex interplay of multimorbidity in patients with COPD and exacerbations increases the risk of poor health outcomes. Due to a lack of specific biomarkers and accurate and cost-effective strategies for differential diagnosis, under- and misdiagnosis are common, as is under- and mistreatment. Consequently, addressing multimorbidity during exacerbations of COPD within a clinical framework, such as syndemic or a treatable traits approach, is required for effective COPD management, and future research is needed to enhance the precision and applicability of these frameworks.
