# Supplementary material for: Bidirectional Clinical Interactions among Exacerbations and Comorbidities in COPD: A Narrative Review
Source: Semin Respir Crit Care Med. 2026 Apr 10;47(3):276–86. doi: 10.1055/a-2835-0340 (PMC13286102; doi:10.1055/a-2835-0340)
Supplement: Supplementary file 1 — Supplementary Material [file 10-1055-a-2835-0340_28489660.pdf]

Table S1 Prevalence ranges in numbers of comorbidities in unclassified risk and high risk of exacerbations.

**Comorbidities**	**Minimum-maximum range (%) of prevalence among studies**	
	Unclassified risk of exacerbations	High risk of exacerbations
Cardiovascular		
- Hypertension	18.2 – 74.9	25.2 – 77.8
- Ischemic heart disease	5.9 – 51.4	4.3 – 38.3
- Heart failure	1.3 – 29.4	1.9 – 32.8
- Coronary artery disease	9.7 – 60.8	14.6 – 21.4
Metabolic		
- Dyslipidemia	36.0 – 70.2	10.1 – 41.2
- Metabolic syndrome	0.6 – 59.4	32.6 – 42.9
- Diabetes Mellitus	1.6 – 40.9	10.4 – 35.8
- Obesity	0.1 – 29.1	0.1 – 29.4
Psychological		
- Anxiety	3.6 – 32.1	18.3 – 46.9
- Depression	7.4 – 57.7	7.0 – 70.1
Cachectic		
- Osteoporosis	6.8 – 31.0	1.1 – 15.8
- Sarcopenia	12.4 – 27.5	16.4 – 48.1
Other		
- Malignancy	1.9 – 13.6	1.7 – 24.1
- Chronic kidney disease	0.6 – 25.8	0.8 – 16.2
- Sleep apnea	6.7 – 16.6	1.3 – 15.0
- Osteoarthritis	19.5 – 69.6	18.8
- Gastro-oesophageal reflux	2.4 – 42.6	1.9 – 15.9

Seminars in Respiratory and Critical Care Medicine | DOI 10.1055/a-2835-0340 | © The Author(s).
